# Pattern and Frequency of Nosocomial Infections in the Pediatric Intensive Care Unit at East Jeddah General Hospital, Saudi Arabia

**DOI:** 10.7759/cureus.47561

**Published:** 2023-10-24

**Authors:** Mohammed A Almazeedi, Hussain A Al Ghadeer, Amani S Bugshan, Hana L Alhrthi, Motaz K Alshuaibi, Hussain H Albarqi, Abdullah M Madkhali, Omar M Maimsh, Sirar A Alali, Ahmed A Al Shams, Danah S Alali, Fatimah A Alhulw, Abdulelah A Alneamah, Mariya A Budris, Khalifah K Alfarhan

**Affiliations:** 1 Pediatrics, East Jeddah General Hospital, Jeddah, SAU; 2 Pediatrics, Maternity and Children Hospital, Al-Ahsa, SAU; 3 Infection Prevention and Control, East Jeddah General Hospital, Jeddah, SAU; 4 Pediatrics, New Medical Center (NMC) Royal Hospital Sharjah, Sharjah, ARE; 5 Pediatrics, King Faisal University, Al-Ahsa, SAU; 6 Pediatrics, Al-Jafar General Hospital, Al-Ahsa, SAU; 7 Pediatrics, Vision Colleges, Riyadh, SAU

**Keywords:** pediatric intensive care unit (picu), saudi arabia, sepsis, vap, clabsi, cauti, nosocomial infection

## Abstract

Background

Because of the use of invasive devices and procedures in critically sick patients, patients in the pediatric intensive care unit (PICU) are particularly vulnerable to nosocomial infections. Although a significant illness may necessitate admission to the PICU, infections can also emerge after admission. Nosocomial infection is a major public health issue related to increased morbidity, death, and healthcare costs. This study aimed to determine the pattern, frequency, and outcomes of nosocomial infections among children who were admitted to the PICU.

Methodology

This retrospective, cross-sectional study was conducted in the pediatric population aged from one month to 14 years old who acquired infections after 48 hours of admission to the PICU at East Jeddah General Hospital, Saudi Arabia from 2021 to 2022. The data were collected from medical and laboratory records.

Results

A total of 51 patients developed 145 nosocomial infections. Central line-associated bloodstream infections (CLABSIs) were the most commonly reported type of nosocomial infections (28.3%). The majority of the isolated organisms (58.7%) were gram-negative, followed by fungal infections (35.1%) and gram-positive organisms (6.2%). The death rate for patients with nosocomial infections was 29.4%. Increased death rates among individuals with CLABSIs and gram-negative isolates were observed to be significantly correlated (p = 0.001).

Conclusions

Our findings suggest that regular surveillance systems were necessary to assess the relationship between these well-known risk variables with PICU, implying that preventing these infections through particular treatments could be cost-effective and contribute to the safety of healthcare systems.

## Introduction

Hospital-acquired infections (HAIs) are a major problem in the medical industry, posing a significant danger to both patients and the healthcare system. Nosocomial infections or HAIs are illnesses caught from a hospital by a patient hospitalized for a cause other than that particular infection or acquired during a hospital visit. They are major sources of morbidity and death in pediatric hospitals [1,2]. Despite the fact that HAIs in pediatric critical care units are a worldwide problem, their prevalence varies substantially from country to country. Because of their immature immune systems, children are more susceptible to illnesses. Young age, mechanical ventilation, sedation, nasogastric tube feeding, prolonged hospitalization, comorbidities, and recurrent aspiration are recognized risk factors for developing nosocomial infections [3-5].

Numerous HAI subtypes exist, including bloodstream infections (BSIs), pneumonia (including ventilator-associated pneumonia (VAP)), urinary tract infections (UTIs), surgical site infections (SSIs), and skin infections [6,7]. According to a study conducted among children who were hospitalized in the pediatric intensive care unit (PICU), out of the 917 blood cultures that were obtained, approximately 11.3% of the cultures grew organisms. Bacterial gram-positive cocci were the most frequently isolated (55.7%), followed by bacterial gram-negative bacilli (31.7%), while fungi (12.5%) accounted for the lowest rate of infections overall. The most frequent bacteria causing BSIs in PICUs, according to other studies, include coagulase-negative *Staphylococcus *[8,9]. Nosocomial infections in children are likely to be more frequent and serious in developing countries due to the malnourished state of patients, relatively limited resources, and improper settings, all of which contribute to morbidities and mortality. The prevalence of HAIs might range from 6.1% to 26% [1,2]. In contrast, developed countries have lower rates of HAIs among infants and children.

In PICUs, according to the National Nosocomial Infection Surveillance System (NNIS) in the United States, there are 14.1% nosocomial infections per 1,000 patients [10]. The incidences in Europe range from 1% in general pediatric wards to 23.6% in PICUs [11]. According to a study conducted in Peru, the incidence of HAIs in PICUs was 19.5% [12] compared to 4.7% [13], 15.6% [14], and 14.7% in Pakistan, Egypt, and the Middle East, respectively. If infection surveillance is combined with efficient infection control programs, the incidence and prevalence of HAIs can be reduced by 32% [15,16]. The prevalence of these HAIs among pediatric patients varies across nations. Data from the Kingdom of Saudi Arabia is scarce.

Hand hygiene is the most important precaution against the transmission of microorganisms in hospitals. This low-cost and simple precaution has been described as being capable of preventing half of nosocomial infections [17]. Low compliance with hand hygiene leads to the emergence of new and different microorganisms by affecting the hospital flora, as well as to an increase in nosocomial infections. International guidelines recommend ensuring hand hygiene with frequent washing with soap and water and rubbing the hands with alcohol-based hand disinfectant. With optimum hand hygiene, the incidence of HAIs is known to be low, and there is a decreased risk of microorganism transmission. However, research into epidemics has noted that compliance is low [18].

## Materials and methods

The retrospective, cross-sectional study was conducted among pediatric patients aged from one month to 14 years old who developed nosocomial infections in the PICU at East Jeddah General Hospital in Saudi Arabia during the course of two years (2021-2022). The non-probability convenience sampling strategy was used to recruit patients. Patients who already had an infection at the time of admission or who developed one within 48 hours of admission were excluded. Medical records and laboratory results were used to obtain data from East Jeddah General Hospital. Positive culture specimens (blood, urine, sputum, and surgical site) for hospitalized patients are stored in a database. The medical records of patients were reviewed to acquire information such as demographic data (age and gender), country, isolated organism, associated device, antibiotic sensitivity, and resistance. The Centers for Disease Control and Prevention (CDC)-identified PICU-acquired nosocomial infections [19]. Infections that began within 48 hours of admission to the PICU were classified as PICU-acquired infections.

For a UTI, a patient must exhibit at least one of the following signs or symptoms without any other known underlying medical conditions: fever (>38°C), urgency, frequency, dysuria, and a positive urine culture with counts of 105 colony-forming units (CFU/mL).

For pneumonia, chest radiographs with new pulmonary infiltrates or the advancement of existing ones were required, along with two of the following signs or symptoms: leukocytosis (>10,000/mm^3^) or leukopenia (4,500/mm^3^), fever (>38°C) or hypothermia (35°C), purulent sputum, and a tracheal aspirate bacterial count of 106 CFU/mL. Bacteremia was defined as the biological evidence of infection, resulting from a positive blood culture.

The data were collected, reviewed, and fed to SPSS version 21 (IBM Corp., Armonk, NY, USA). All statistical methods used were two-tailed with an alpha level of 0.05 considering significance if p-values were less than or equal to 0.05. Patient ID (MRN) was used for matching cases with reported infections. Descriptive analysis was done by prescribing frequency distribution and percentage for study variables including patient demographic and medical data per case. Likewise, the number of HAIs reported for all cases was tabulated and graphed, including types, isolated organisms, and antibiotic resistance. Further, clinical outcome among infected cases was graphed. Cross-tabulation for showing factors associated with in-hospital mortality among pediatric patients with HAIs in the PICU was performed using Pearson’s chi-square test for significance and exact probability test if there were small frequency distributions.

## Results

A total of 51 patients developed 145 HAIs with an incidence rate of 35 per 1,000-person days. Patients’ ages ranged from one month to 13 years with a mean age of 3.5 ± 4.2 years. In total, 32 (62.7%) patients had comorbidities (Table 1).

**Table 1:** Personal data of study patients with nosocomial infections in the pediatric intensive care unit (n = 51).

Personal data	N	%
Age in years		
<1	21	41.2%
1–4	16	31.4%
5–9	6	11.8%
10–14	8	15.7%
Mean ± SD	3.5 ± 4.2	
Comorbidity		
Yes	32	62.7%
No	19	37.3%

**Figure 1:**
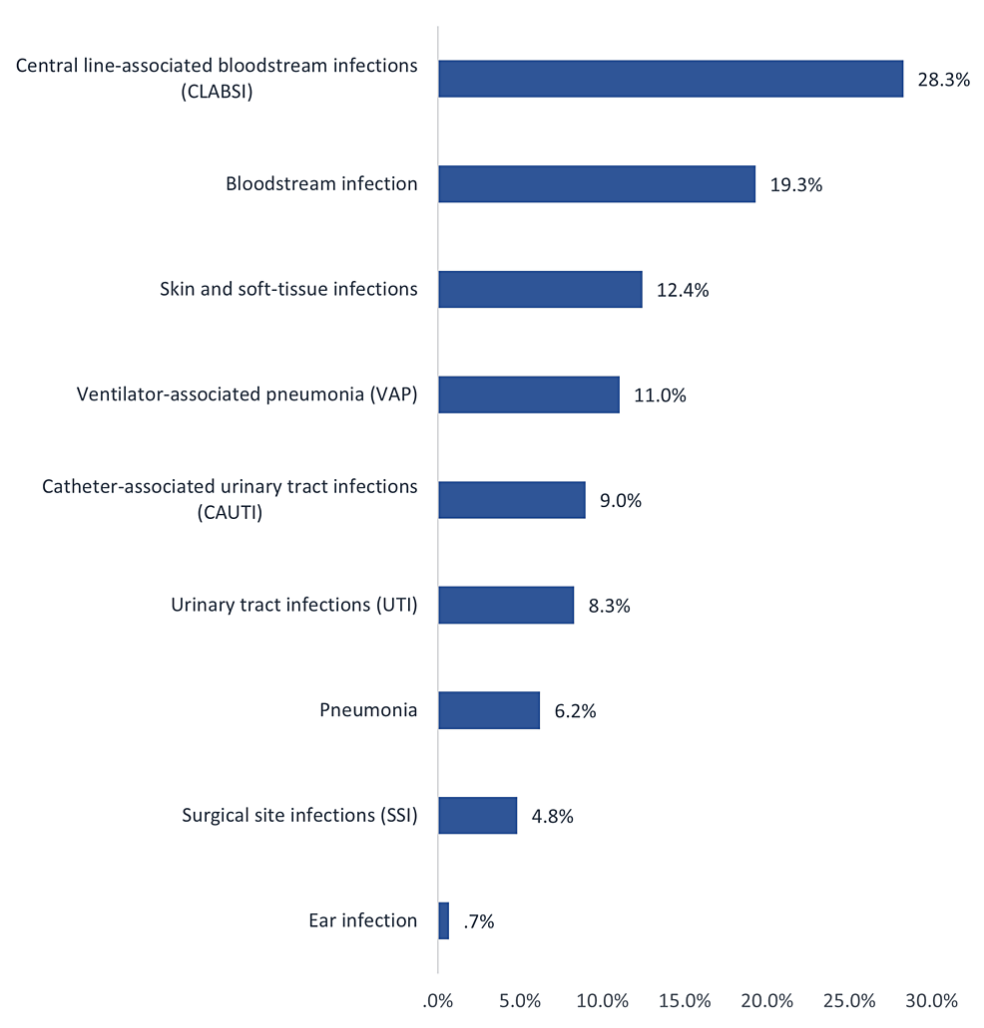
Types of nosocomial infections in the pediatric intensive care unit (n = 145).

Figure 1 shows the types of HAIs in the PICU at East Jeddah General Hospital. The most reported types were central line-associated bloodstream infections (CLABSIs) (28.3%; 41), followed by BSIs (19.3%; 28), skin and soft-tissue infections (12.4%; 18), VAP (11%; 16), catheter-associated urinary tract infections (CAUTIs) (9%; 13), UTIs (8.3%; 12), and pneumonia (6.2%; 9). Regarding the obtained samples, 31 were from the blood through a peripheral line, 29 were from the blood through the central line, 26 were urine samples, 24 were wound swabs, 16 were tracheal aspirates, nine were sputum samples, nine were blood from a central and peripheral line, and one was an ear swab sample.

Table 2 presents the isolated organisms among HAIs in the PICU at East Jeddah General Hospital. The most commonly isolated organisms were gram-negative organisms (58.7%; 85) mainly *Klebsiella pneumoniae* (35.2%), *Escherichia coli* (9%), and *Pseudomonas aeruginosa* (6.9%). A total of 51 (35.1%) organisms were yeasts mainly *Candida parapsilosis* (12.4%; 18), *Candida tropicalis* (11.7%; 17), and *Candida albicans* (4.1%; 6). Only nine (6.2%) organisms were gram-positive mainly *Staphylococcus aureus* (5.5%; 8), and 1 case had *Streptococcus pyogenes* (group A).

**Table 2:** Isolated organisms of nosocomial infections in the pediatric intensive care unit (n = 145).

Isolated organism	N	%
Fungi		
Candida parapsilosis	18	12.4%
Candida tropicalis	17	11.7%
Candida albicans	6	4.1%
Candida lusitaniae	6	4.1%
Candida famata	3	2.1%
Yeast	1	0.7%
Total	51	35.1%
Gram-negative		
Klebsiella pneumoniae	51	35.2%
Escherichia coli	13	9.0%
Pseudomonas aeruginosa	10	6.9%
Acinetobacter baumanii	6	4.1%
Multidrug-resistant *Stenotrophomonas maltophilia*	2	1.4%
Citrobacter freundii	1	0.7%
Enterobacter cloacae	1	0.7%
Proteus mirabilis	1	0.7%
Total	85	58.7%
Gram-positive		
Staphylococcus aureus	8	5.5%
*Streptococcus pyogenes* (group A)	1	0.7%
Total	9	6.2%

**Figure 2:**
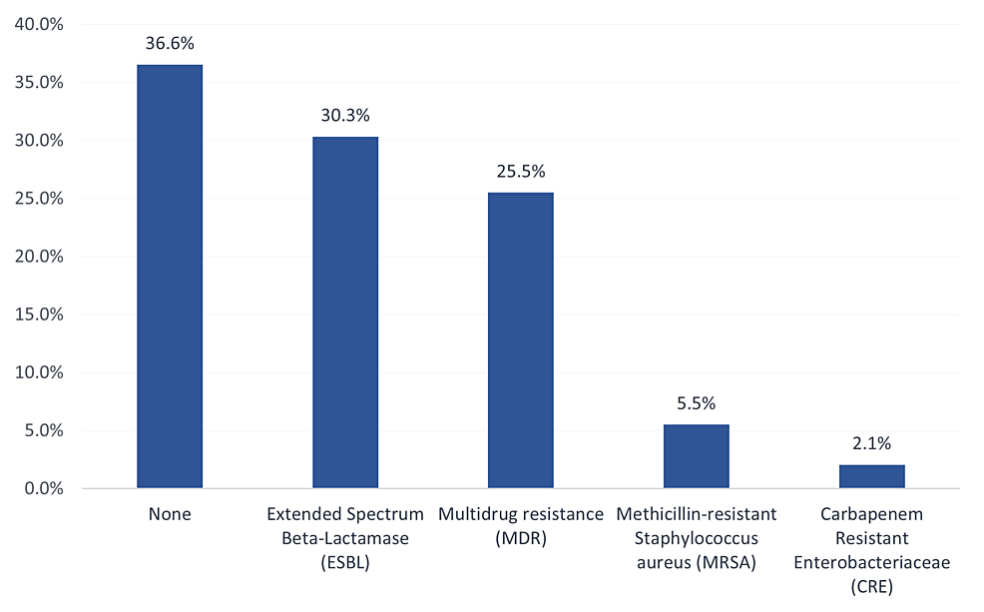
Antibiotic resistance among isolated organisms of nosocomial infections in the pediatric intensive care unit (n = 145).

Figure 2 shows antibiotic resistance among isolated organisms from HAIs in the PICU. A total of 44 (30.3%) samples showed extended-spectrum beta-lactamase resistance, 37 (25.5%) showed multidrug resistance, eight (5.5%) were resistant to methicillin-resistant Staphylococcus aureus, and three (2.1%) were carbapenem-resistant Enterobacteriaceae. A total of 53 (36.6%) had no resistance to antibiotics.

**Figure 3:**
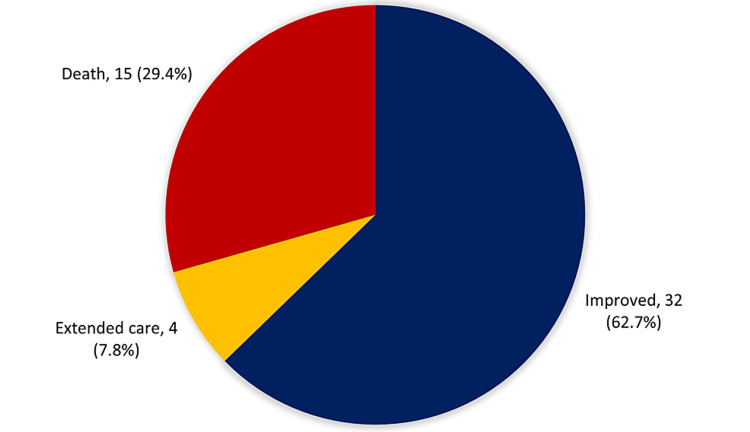
Clinical outcomes among patients with nosocomial infections in the pediatric intensive care unit (n = 51).

Figure 3 shows the clinical outcomes among patients with HAIs in the PICU (n = 51). The vast majority of the patients (62.7%; 32) improved, four (7.8%) needed extended care, and 15 (29.4%) died.

Table 3 presents the factors associated with in-hospital mortality among pediatric patients with HAIs in the PICU. Overall, 42.9% of infants below the age of one year with HAIs died compared to 12.5% of patients aged 10-14 years with recorded statistical significance (p = 0.048). Additionally, the highest in-hospital mortality rate was reported among patients who had CLABSIs (48.8%), BSIs (46.4%), and UTIs (41.7%) compared to none who had ear infections and SSIs (p = 0.028). Likewise, 56.9% of infants infected with fungi died compared to 25.9% of others infected with gram-negative bacteria and none infected with gram-positive bacteria (p = 0.001).

**Table 3:** Factors associated with in-hospital mortality among pediatric patients with nosocomial infections in the pediatric intensive care unit. P: exact probability test; *: p <0.05 (significant)

Factors	Outcomes				P-value
	Died		Survived		
	No	%	No	%	
Age in years (n = 51)					0.048*
<1	9	42.9%	12	57.1%	
1–4	5	31.3%	11	68.8%	
5–9	0	0.0%	6	100.0%	
10–14	1	12.5%	7	87.5%	
Comorbidity (n = 51)					0.708
Yes	10	31.3%	22	68.8%	
No	5	26.3%	14	73.7%	
Type of hospital-acquired infections (n = 145)					0.028*
Bloodstream infections	13	46.4%	15	53.6%	
Catheter-associated urinary tract infections	5	38.5%	8	61.5%	
Central line-associated bloodstream infections	20	48.8%	21	51.2%	
Ear infections	0	0.0%	1	100.0%	
Pneumonia	3	33.3%	6	66.7%	
Skin and soft-tissue infections	1	5.6%	17	94.4%	
Surgical site infections	0	0.0%	7	100.0%	
Urinary tract infections	5	41.7%	7	58.3%	
Ventilator-associated pneumonia	4	25.0%	12	75.0%	
Type of isolated organism (n = 145)					0.001*
Fungal	29	56.9%	22	43.1%	
Gram-negative	22	25.9%	63	74.1%	
Gram-positive	0	0.0%	9	100.0%	

## Discussion

HAIs in PICUs are a significant concern as they can cause significant morbidity and mortality in critically ill children [20,21]. Critically ill children often require invasive devices, such as central venous catheters, urinary catheters, and endotracheal tubes, which increase their risk of acquiring infections [22]. These infections can have a significant impact on patient outcomes, hospital length of stay, and costs [23]. Preventing HAIs in PICUs is crucial.

This study aimed to determine the pattern and frequency of HAIs in the PICU at East Jeddah General Hospital, Jeddah, Saudi Arabia. The study results showed that 51 developed 145 HAIs with an incidence rate of 35 per 1,000-person days. Patients’ ages ranged from one month to 13 years with a mean age of 3.5 ± 4.2 years. Overall, 32 (62.7%) patients had comorbidities. Regarding the types of reported HAIs, BSIs including CLABSIs were the most frequent, followed by skin and soft-tissue infections, pneumonia, and UTIs. The incidence rate of nosocomial infections per 1,000 patient days for PICUs was documented by the NNIS in the United States at 14.1 [9]. This rate is almost half that of the current study. In India, the reported infection rate and incidence density of HAIs in the PICU were 19.3/100 patients and 21/1,000 patient days, respectively. In Europe, the incidence of HAIs varied from 1% in general pediatric wards to up to 23.6% in PICUs [11]. Patients with HAIs had significantly longer average stays and higher mortality rates (25 vs. 7 days, p = 0.0001 and 50% vs. 27.8% p = 0.005, respectively) [24]. The most common nosocomial infections in PICUs are BSIs, pneumonia (including VAP), and UTIs, followed by enteric, surgical site, and skin infections [7,8]. A minimum of one or more HAIs were reported by Kepenekli et al. [25]. Lower respiratory tract infections were most frequently reported. In addition, 35 out of 466 patients had 49 episodes of nosocomial infections, according to Ahirrao et al. [26]. The incidence rate of nosocomial infections was 10.51 per 100 admissions. The incidence density of nosocomial infections was 19.37 per 1,000 patient days. Alotaibi et al. [27] reported a lower incidence in Saudi Arabia, with only 3% of patients acquiring nosocomial infections in 684 admissions in 2012. The most common occurrence was found in the first year of life (10 patients). Respiratory infections were the most commonly reported type of infection, followed by urinary infections.

Regarding isolated organisms, the study found that gram-negative organisms (more than half of HAIs) were the most common, with *Klebsiella pneumoniae* being the most isolated organism, followed by *Escherichia coli* and* Pseudomonas aeruginosa* (6.9%). One-third of the HAIs were caused by fungi, primarily *Candida parapsilosis*, *Candida tropicalis*, and *Candida albicans*. Only nine organisms were gram-positive, the majority of which were *Staphylococcus aureus*, with one case of *Streptococcus pyogenes *(group A).

According to a review of the literature, gram-positive cocci accounted for 55.7% of the blood isolates obtained over a one-year period, followed by gram-negative bacilli (GNB) at 31.7% and yeast at 12.5%. Coagulase-negative staphylococci were the most common organisms isolated, followed by *Candida* spp., *Klebsiella pneumoniae*, and *Acinetobacter *spp. in this order [28]. Other studies have found that the most common pathogen causing BSIs in PICUs is coagulase-negative *Staphylococcus *[9,10]. According to Singhi et al. [29], gram-negative organisms were the most common, with *Klebsiella pneumoniae* (20.1%), *Enterobacter *spp. (16.6%), and *Acinetobacter *spp. (8.6%) being the most common. According to Alotaibi et al. [27], *Klebsiella pneumoniae* and *Pseudomonas aeruginosa* were the pathogens most frequently isolated from respiratory infections, despite the fact that *Klebsiella* and *Candida *were isolated from UTIs. *Candida *and *Klebsiella pneumoniae* were the most typical pathogens responsible for septicemia.

Considering the clinical outcomes, this study revealed that the in-hospital mortality rate among study patients did not exceed one-third and the vast majority of the patients (about two-thirds) improved. The overall mortality attributable to pediatric HAIs has been estimated at 11% [8]. Becerra et al. [8] found that the mortality of HAIs was 38%, notably greater than that reported from PICUs of developed countries (7.7-10%) [15,30]. Higher mortality was reported among young age patients and those with BSIs and UTIs. Moreover, fungal infections were associated with the highest mortality rate in this study.

This study has certain limitations, including the fact that it is a retrospective study, which makes it difficult to investigate other lab-related studies to be correlated clinically and recall potential biases in data collection. Second, there is a paucity of evidence on re-intubation, which may be crucial for VAP. Furthermore, we were unable to determine attributable mortality risk factors that may have impacted the results. Although this study has certain limitations, it is one of the few studies conducted in our area and will help us in strategizing our infection control measures. Finally, it is recommended to conduct further prospective studies for more accurate data.

## Conclusions

This study estimated a high incidence of HAIs, mainly BSIs, pneumonia, and UTIs, among pediatric patients in the PICU. Gram-negative organisms were the most isolated followed by fungi. The in-hospital mortality rate was less than one-third of the patients but most patients improved with the highest mortality associated with young age and fungal infections. Active surveillance of healthcare-associated infections is essential for effective management. It is important to follow infection prevention protocols, including proper hand hygiene, proper insertion and maintenance of invasive devices, and appropriate use of antimicrobials. Implementing these measures can help reduce the risk of HAIs and improve patient outcomes in PICUs.

## References

[1] Abdel-Fattah MM (2005). Surveillance of nosocomial infections at a Saudi Arabian military hospital for a one-year period. Ger Med Sci.

[2] Almuneef MA, Memish ZA, Balkhy HH, Hijazi O, Cunningham G, Francis C (2006). Rate, risk factors and outcomes of catheter-related bloodstream infection in a paediatric intensive care unit in Saudi Arabia. J Hosp Infect.

[3] Asembergiene J, Gurskis V, Kevalas R, Valinteliene R (2009). Nosocomial infections in the pediatric intensive care units in Lithuania. Medicina (Kaunas).

[4] El-Sahrigy SA, Shouman MG, Ibrahim HM (2019). Prevalence and anti-microbial susceptibility of hospital acquired infections in two pediatric intensive care units in Egypt. Open Access Maced J Med Sci.

[5] Mansour E, Bendary S (2012). Hospital acquired pneumonia in critically ill children: incidence, risk factors, outcome and diagnosis with insight on the novel diagnostic technique of multiplex polymerase chain reaction. Egypt J Med Human Genet.

[6] Patra PK, Jayashree M, Singhi S, Ray P, Saxena AK (2007). Nosocomial pneumonia in a pediatric intensive care unit. Indian Pediatr.

[7] Grohskopf LA, Sinkowitz-Cochran RL, Garrett DO (2002). A national point-prevalence survey of pediatric intensive care unit-acquired infections in the United States. J Pediatr.

[8] Becerra MR, Tantaleán JA, Suárez VJ, Alvarado MC, Candela JL, Urcia FC (2010). Epidemiologic surveillance of nosocomial infections in a pediatric intensive care unit of a developing country. BMC Pediatr.

[9] Richards MJ, Edwards JR, Culver DH, Gaynes RP (1999). Nosocomial infections in pediatric intensive care units in the United States. National Nosocomial Infections Surveillance System. Pediatrics.

[10] Elward AM, Fraser VJ (2006). Risk factors for nosocomial primary bloodstream infection in pediatric intensive care unit patients: a 2-year prospective cohort study. Infect Control Hosp Epidemiol.

[11] Raymond J, Aujard Y (2000). Nosocomial infections in pediatric patients: a European, multicenter prospective study. European Study Group. Infect Control Hosp Epidemiol.

[12] Haque A, Bano S (2009). Clinical profile and outcome in a paediatric intensive care unit in Pakistan. J Coll Physicians Surg Pak.

[13] Lukas S, Hogan U, Muhirwa V (2016). Establishment of a hospital-acquired infection surveillance system in a teaching hospital in Rwanda. Int J Infect Control.

[14] Lodha R, Natchu UC, Nanda M, Kabra SK (2001). Nosocomial infections in pediatric intensive care units. Indian J Pediatr.

[15] Raymond J (2000). [Epidemiology of nosocomial infections in pediatrics]. Pathol Biol (Paris).

[16] Porto JP, Mantese OC, Arantes A, Freitas C, Gontijo Filho PP, Ribas RM (2012). Nosocomial infections in a pediatric intensive care unit of a developing country: NHSN surveillance. Rev Soc Bras Med Trop.

[17] Siegel JD (2007). 2007 Guideline for Isolation Precautions: Preventing Transmission of Infectious Agents in Healthcare Settings. https://www.google.com/url?sa=t&rct=j&q=&esrc=s&source=web&cd=&cad=rja&uact=8&ved=2ahUKEwivlNKKoumBAxXasFYBHZnqAnEQFnoECBUQAQ&url=https%3A%2F%2Fwww.cdc.gov%2Finfectioncontrol%2Fpdf%2Fguidelines%2Fisolation-guidelines-H.pdf&usg=AOvVaw3b02eCeSvTdGpmb0MeGCQV&opi=89978449.

[18] Aktar F, Tekin R, Güneş A (2016). Determining the independent risk factors and mortality rate of nosocomial infections in pediatric patients. Biomed Res Int.

[19] Horan TC, Andrus M, Dudeck MA (2008). CDC/NHSN surveillance definition of health care-associated infection and criteria for specific types of infections in the acute care setting. Am J Infect Control.

[20] Guzman-Cottrill JA, Kirby A (2014). Healthcare-associated infections in the pediatric intensive care unit. J Pediatr Intensive Care.

[21] Tweddell S, Loomba RS, Cooper DS, Benscoter AL (2019). Health care-associated infections are associated with increased length of stay and cost but not mortality in children undergoing cardiac surgery. Congenit Heart Dis.

[22] Deptuła A, Trejnowska E, Ozorowski T, Hryniewicz W (2015). Risk factors for healthcare-associated infection in light of two years of experience with the ECDC point prevalence survey of healthcare-associated infection and antimicrobial use in Poland. J Hosp Infect.

[23] Briassoulis P, Briassoulis G, Christakou E (2021). Active surveillance of healthcare-associated infections in pediatric intensive care units: multicenter ECDC HAI-net ICU protocol (v2.2) implementation, antimicrobial resistance and challenges. Pediatr Infect Dis J.

[24] Gupta A, Kapil A, Lodha R (2011). Burden of healthcare-associated infections in a paediatric intensive care unit of a developing country: a single centre experience using active surveillance. J Hosp Infect.

[25] Kepenekli E, Soysal A, Yalindag-Ozturk N (2015). Healthcare-associated infections in pediatric intensive care units in Turkey: a national point-prevalence survey. Jpn J Infect Dis.

[26] Ahirrao VS, Mauskar A, Ravi T (2017). Incidence of nosocomial infection in the pediatric intensive care unit of a teaching hospital delivering tertiary level care. Int J Contemp Pediatr.

[27] Alotaibi MG, Rahman S, Al-Shalaan MA (2015). Frequency of nosocomial infections in pediatric intensive care unit at King Abdulaziz Medical City, Riyadh, Saudi Arabia. J Infect Dis Ther.

[28] Wattal C, Oberoi J (2012). Infections in pediatric intensive care units (PICU). Indian J Pediatr.

[29] Singhi S, Ray P, Mathew JL, Jayashree M (2008). Nosocomial bloodstream infection in a pediatric intensive care unit. Indian J Pediatr.

[30] Urrea M, Pons M, Serra M, Latorre C, Palomeque A (2003). Prospective incidence study of nosocomial infections in a pediatric intensive care unit. Pediatr Infect Dis J.

